# Parameter Identifiability and Redundancy in a General Class of Stochastic Carcinogenesis Models

**DOI:** 10.1371/journal.pone.0008520

**Published:** 2009-12-31

**Authors:** Mark P. Little, Wolfgang F. Heidenreich, Guangquan Li

**Affiliations:** 1 Department of Epidemiology and Public Health, Imperial College, London, United Kingdom; 2 Institut für Strahlenschutz, Helmholtz Zentrum München, Neuherberg, Germany; University of Nottingham, United Kingdom

## Abstract

**Background:**

Heidenreich *et al.* (*Risk Anal* 1997 **17** 391–399) considered parameter identifiability in the context of the two-mutation cancer model and demonstrated that combinations of all but two of the model parameters are identifiable. We consider the problem of identifiability in the recently developed carcinogenesis models of Little and Wright (*Math Biosci* 2003 **183** 111–134) and Little *et al.* (*J Theoret Biol* 2008 **254** 229–238). These models, which incorporate genomic instability, generalize a large number of other quasi-biological cancer models, in particular those of Armitage and Doll (*Br J Cancer* 1954 **8** 1–12), the two-mutation model (Moolgavkar *et al. Math Biosci* 1979 **47** 55–77), the generalized multistage model of Little (*Biometrics* 1995 **51** 1278–1291), and a recently developed cancer model of Nowak *et al.* (*PNAS* 2002 **99** 16226–16231).

**Methodology/Principal Findings:**

We show that in the simpler model proposed by Little and Wright (*Math Biosci* 2003 **183** 111–134) the number of identifiable combinations of parameters is at most two less than the number of biological parameters, thereby generalizing previous results of Heidenreich *et al.* (*Risk Anal* 1997 **17** 391–399) for the two-mutation model. For the more general model of Little *et al.* (*J Theoret Biol* 2008 **254** 229–238) the number of identifiable combinations of parameters is at most 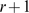 less than the number of biological parameters, where 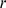 is the number of destabilization types, thereby also generalizing all these results. Numerical evaluations suggest that these bounds are sharp. We also identify particular combinations of identifiable parameters.

**Conclusions/Significance:**

We have shown that the previous results on parameter identifiability can be generalized to much larger classes of quasi-biological carcinogenesis model, and also identify particular combinations of identifiable parameters. These results are of theoretical interest, but also of practical significance to anyone attempting to estimate parameters for this large class of cancer models.

## Introduction

Models for complex biological systems may involve a large number of parameters. In principle it may well be that some of these parameters may not be observed, or be possible to be derived from observed data via regression techniques. Such parameters are said to be unidentifiable or non-identifiable, the remaining parameters being identifiable.

There is a substantial literature on identifiability in stochastic models in various contexts [1], [2], [3]. Catchpole and Morgan [3] considered identifiability and parameter redundancy and the relations between them in a general class of (exponential family) models. Catchpole and Morgan [3] defined a set of model parameters in an exponential family model to be *redundant* if the likelihood can be written using a strictly smaller parameter vector; otherwise they are *irredundant*. Rothenberg [1], Jacquez and Perry [4] and Catchpole and Morgan [3] also defined a notion of *local identifiability*, to mean that within a neighbourhood of each set of parameter values the likelihood differs for at least some data points. This notion has been extended by Little *et al.* [5] to *gradient weak local identifiability* and *weak local identifiability*. Little *et al.* [5] defined a set of parameters to be *weakly locally identifiable* if the maxima of the likelihood are isolated; they defined parameters to be *gradient weakly locally identifiable* if the turning points (those for which the likelihood derivative with respect to the parameters is zero) are isolated. The results obtained by Little *et al.* [5] (Corollary 2 (ii) and the subsequent Remark (ii)), show that, subject to some regulatory conditions, the number of locally identifiable or (gradient) weakly locally identifiable parameter combinations is equal to the rank of the Hessian matrix, or equivalently the rank of the Fisher information matrix. The notions of identifiability in stochastic models [1], [2], [3], [5], within which framework this paper is set, should be contrasted with the consideration of identifiablity in non-stochastic settings considered by some [4], [6], [7].

Heidenreich [8] and Heidenreich *et al.* [9] considered parameter identifiability in the context of the two-mutation cancer model [10] and demonstrated that of the five biological parameters in the model, on the basis of the cancer hazard function only three could be identified. [It should be noted that given extra information, for example on numbers and sizes of intermediate cell compartment clones, there is information on an additional parameter.]

In this paper we consider the problem of identifiability in recently developed carcinogenesis models of Little and Wright [11] and Little *et al.* [12]. These models generalize a large number of other quasi-biological cancer models, in particular those of Armitage and Doll [13], the two-mutation model [10], the generalized multistage model of Little [14], and a recently developed cancer model of Nowak *et al.* [15] that incorporates genomic instability. We shall show that via a specific reparameterization, in the simpler model proposed by Little and Wright [11] in principle combinations of all but two of the model parameters are identifiable, thereby generalizing previous results of Heidenreich [8] and Heidenreich *et al.* [9] for the two-mutation cancer model. For the more general model of Little *et al.* [12] combinations of all but 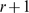 of the model parameters are identifiable, where 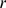 is the number of destabilization types, thereby also generalizing all these results. We also identify particular forms of identifiable parameters.

## Methods

### Parameter Identifiability in the Context of a Stochastic Cancer Model with Genomic Instability

We consider the problem of parameter identifiability in a particular class of stochastic cancer models, those of Little and Wright [11] and Little *et al.* [12]. The ideas used are similar to those employed by Heidenreich *et al.* [9], in particular the use of Cauchy's method of characteristics. We shall assume throughout this section that this model is embedded in a member of the exponential family so that the log-likelihood is given by 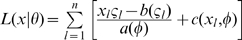 where the natural parameters 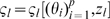 are functions of the model parameters 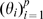 and some auxiliary data 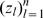, but that the scaling parameter 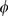 is not. We shall assume that the 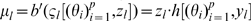, where 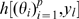 is the cancer hazard function, and that the 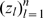 are all non-zero. This is generally the case, in particular when cohort data are analysed using Poisson regression models, e.g., as in Little and Wright [11] or Little and Li [16]. By the remarks following Corollary 2 of Little *et al.* [5], proving weak local identifiability of a subset of cardinality 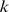 of the biological parameters 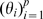 is equivalent to showing that for this subset of parameters 
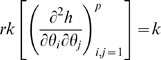
.

**Figure 1:**
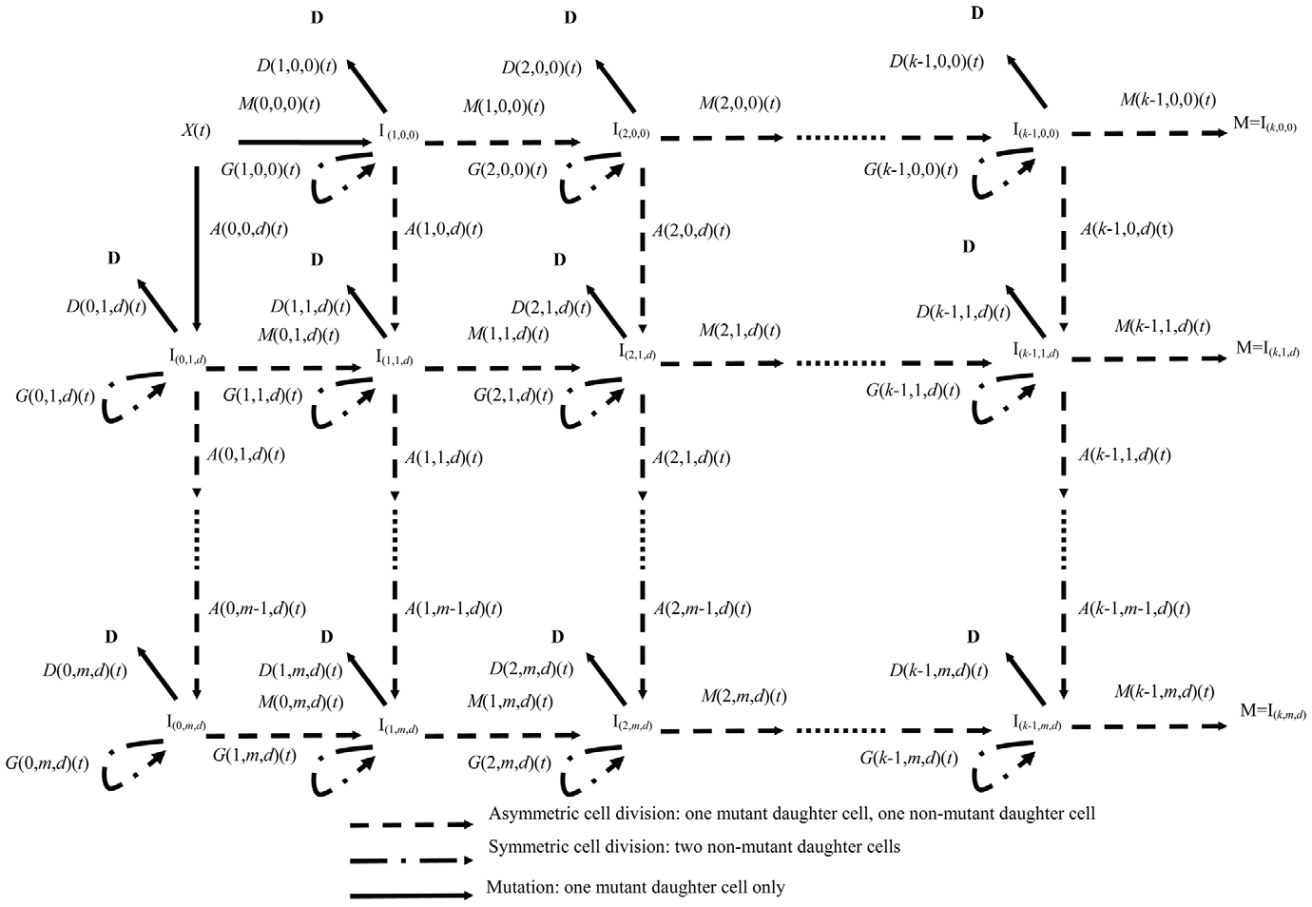
Diagram of cancer model with 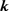 cancer-stage mutations and 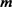 destabilizing mutations, as in [12].

**Figure 2:**
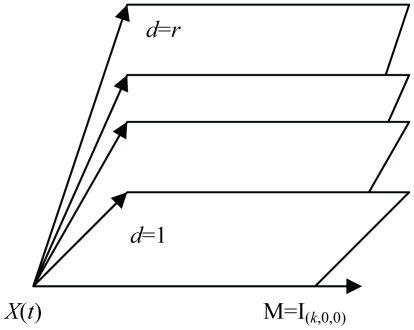
Destabilizing-mutation planes in model, each plane with structure of Figure 1, as in [12].

The model of Little *et al.* [12], generalizing that of Little and Wright [11], which in turn generalizes the model of Little [14], assumes that cells can acquire up to 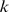 successive cancer-stage mutations, and any of 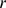 (mutually exclusive) types of destabilization mutation(s). Cells become malignant when *k* cancer-stage mutations have occurred, no matter how many destabilizing mutations there have been. Once a cell has acquired a destabilizing mutation of type 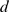 (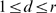), it and its daughter cells can acquire up to 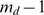 further destabilizing mutations of the same type. We define 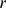 to be the multiplicity of destabilization mutation types. It is to be expected that the more destabilizing mutations cells acquire of each type, the higher the cancer stage mutation rate is, but this is not intrinsic to the model. We write 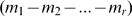 as the *signature of the destabilizing mutation types*. We habitually describe this model as of type 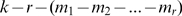 for short. The model is illustrated schematically in Figures 1 and 2. Table 1 lists the biological parameters that are used in the model, and their multiplicity.

**Table 1:** The number of biological parameters in a model with 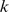 cancer stages, 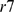 types of GI and 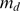 (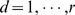) levels of destabilizations.

Model parameter descriptions	Model parameters	Number of such parameters in the model
Stem cell population number	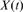	1
Growth rate	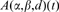	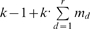
Death/differentiation rate	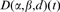	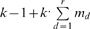
Cancer-stage mutation rate	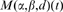	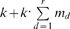
Destabilizing mutation rate	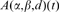	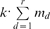
**Total**		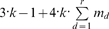

Cells at different stages of the process are labelled by 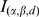, where the first subscript, 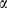, represents the number of cancer stage mutations that the cell has accumulated, the second subscript, 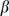, represents the number of destabilizing mutations acquired, their type being given by the third subscript, 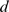. At all stages other than 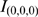, cells are allowed to divide symmetrically or differentiate (or undergo apoptosis) at rates 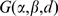 and 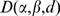, respectively. Each cell can divide into an equivalent daughter cell and another cell with an extra cancer stage mutation at rate 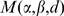. Likewise, cells can also divide into an equivalent daughter cell and another cell with an additional destabilizing mutation of type 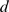 at rate 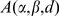. The model assumes that there are 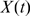 susceptible stem cells at age 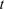. Further details on derivation of the hazard function are given in the paper of Little *et al.* [12].

## Results

In Text S1 Section B we derive the hazard function and show that it can be written in terms of certain combinations of the biological parameters given in Table 1. From equations (B12)–(B16) in Text S1 Section B it is seen that the characteristics and 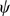 are governed by certain parameter combinations. Table 2 summarizes the maximum number of identifiable parameter combinations and their forms associated with each cell compartment. The maximum number of identifiable parameters associated with each destabilization zone, 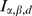, are 4 when 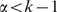 and 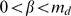; 4 when 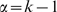 and 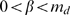; 3 when 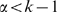 and 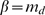 and 2 when 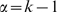 and 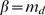. The function 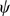 is governed by at most 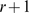 parameter combinations. Therefore, we have shown that the hazard function 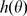 can be written as 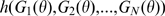 for some scalar functions 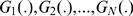, where 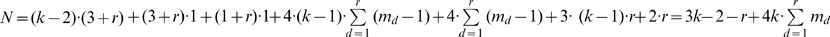 (Table 2). Assuming that the cancer model is embedded in a member of the exponential family (in the sense outlined in Text S1 Section C) the same will be true of the total log-likelihood 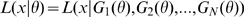. By means of the Chain Rule we obtain 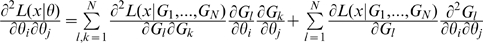, so that the Fisher information matrix is given by
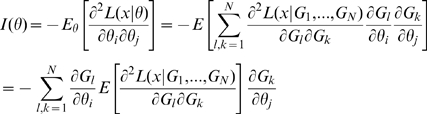
(1)which therefore has rank at most 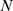. A similar argument shows that if one were to reparameterise (via some invertible 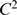 mapping 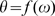) then the embedded log-likelihood 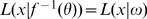 associated with 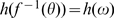 must also have Fisher information matrix of rank at most 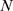. By Theorems 1 and 3 of Catchpole and Morgan [3], for this embedded exponential family model therefore there can be at most 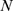 irredundant parameters. Therefore, of the theoretically available 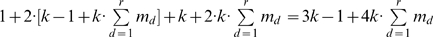 biological parameters (Table 1), at most 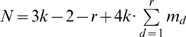 parameter combinations are identifiable, indicating a minimum of 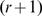 parameter redundancies in the model. Also, from the results obtained by Little *et al.* [5] (Corollary 2 (ii) and the subsequent Remark (ii)), subject to some regulatory conditions, the number of locally identifiable or (gradient) weakly locally identifiable parameter combinations is equal to the rank of the Fisher information matrix, so 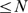. For example, in the case of the familiar two-mutation model [10], with 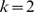, 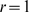, 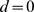 and 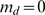, there are 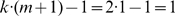 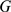
's (namely 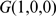), 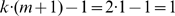 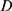
's (namely 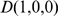), 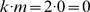 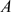
's, 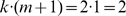 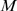
's (namely 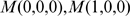), and a single 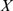, giving a total of five biological parameters. It is known from the results of Heidenreich *et al.* [8], [9] that for the two-mutation model only three combinations of these are estimable, i.e., that there are two redundancies, precisely in agreement with the result given here for 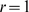. This result therefore precisely generalizes the results and approach of Heidenreich *et al.* [8], [9]. Unfortunately, analytical methods for proving that precisely this number of parameters are estimable, including some recently outlined [17], cannot be used for the model considered here. Nevertheless, we conjecture that in fact precisely this number of parameters are estimable, so that the upper bound on the number of estimable parameter combinations that we have proved above is in fact sharp. This is supported by numerical evaluation of the Hessian in a couple of example cases, which we now outline.

**Table 2:** Parameter combinations associated with each cell compartment. The forms of these combinations are extracted from equations (B12)–(B16) in Text S1.

Compartment 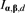	Number of such compartments	Forms of identifiable parameter combinations	Maximum number of identifiable parameter combinations	Total maximum number of identifiable parameter combinations
Principal axis (non-destabilization) 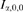 ( 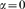 , 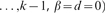				
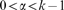	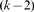	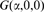 , 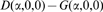 , 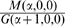 , 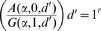	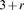	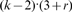
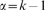	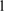	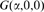 , 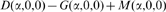 , 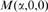 , 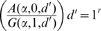	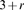	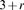
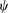 ( 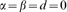 )	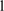	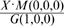 , 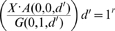	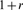	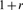
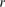 destabilization zones ( 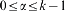 , 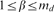 , 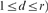				
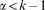 , 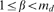	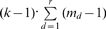	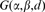 , 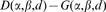 , 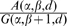 , 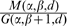	4	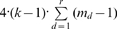
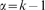 , 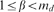	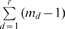	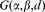 , 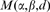 , 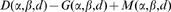 , 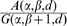	4	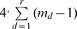
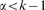 , 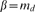	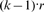	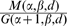 , 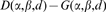 , 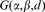	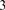	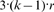
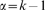 , 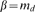	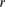	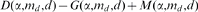 , 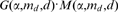	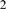	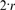
**Total**				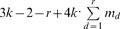

### Numerical Evaluation of Hessian and Determination of Its Rank

That there are likely to be exactly this number of estimable parameters is supported by numerical evaluation of the Hessian matrix of the hazard function. We make use of the solution of the system of ordinary differential equations defining the Hessian, outlined in Text S1 Section D. We will show in two cases that the Hessian has rank two less than the number of biological parameters, 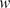. By the above-mentioned results of Catchpole and Morgan [3] and Little *et al* [5] this suggests that precisely 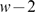 parameters are (gradient) weakly locally identifiable. In order to show that the Hessians are of rank two less than the number of biological parameters, 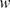, we evaluate the eigenvalues of the Hessian matrix, and establish that the smallest eigenvalue among the 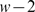 largest eigenvalues in absolute value exceeds the likely magnitude of the error by at least an order of magnitude. We know the likely size of the error in numerical evaluations of each element, 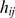, of the Hessian from the Boerlisch-Stoer integrator that is employed, namely 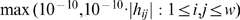 (**bsstep** routine, Press *et al.* [18], p.722). It is known that if two symmetric matrices 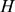 and 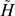 have eigenvalues 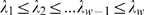 and 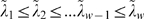 then 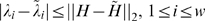, where 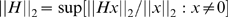 [19](p.396). Since the approximate Hessian that we calculate, 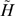, differs from the true Hessian, 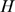, by an amount 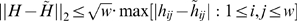, we know that:
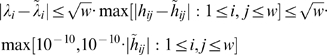
(2)There is also the issue of numerical roundoff error in the QR algorithm (Numerical Algorithms Group (NAG) routine **F02FAF** [20]) used to compute eigenvalues. If we write now 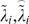 for the true and approximate eigenvalues associated with the approximate Hessian, 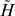, this is known to be bounded by:
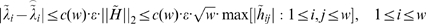
(3)where 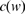 is a modestly increasing function of the dimension, 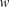, of the approximate Hessian 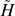 and 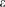 is the machine precision [19](Chapter 8). Since the machine precision (in double precision) is of the order 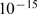 this expression (3) will be dominated by the error associated with the approximation to the Hessian, given by expression (2).

We evaluated the Hessian matrix for a model with three cancer-stage mutations and one destabilizing mutation, and a model with two cancer-stage mutations and one destabilizing mutation; log-normal perturbations of all parameters were performed, assuming a geometric standard deviation (GSD) of 4, centred on models with cancer-stage mutation rates of 4.0×10^−3^ year^−1^, destabilizing mutation rates of 3.0×10^−3^ year^−1^, intermediate cell proliferation rates of 1.0×10^−1^ year^−1^, and intermediate cell death rates of 5.0×10^−1^ year^−1^. For each of 1000 random sets of parameters we evaluated the Hessian by numerical integration, as outlined in Text S1 Section D. We calculated the eigenvalues of the Hessian using the QR algorithm, specifically the NAG FORTRAN subroutine **F02FAF** [20]. For each model we selected the set of random parameters for which the ratio of minimum to maximum among the 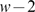 largest eigenvalues (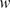 being the number of biological parameters) in absolute value was greatest. These are given in Tables 3 and 4, for the three-stage and two-stage models, respectively. The associated eigenvalues are given in Table 5. The absolute value of the 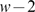
th smallest eigenvalue associated with each set exceeds the error bound (2) by at least an order of magnitude in each case. This strongly suggests that the Hessians calculated for these two examples really are of rank 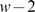 for each model.

**Table 3:** Example coefficients of model with three cancer stage mutations and one destabilizing mutation.

Coefficient	Value
*G*(1,0,0)	8.64714335947694×10^−2^
G(2,0,0)	1.06188950764276×10^−3^
*D*(1,0,0)	4.25556779736062×10^−2^
*D*(2,0,0)	2.68975909218019×10^−1^
*M*(0,0,0)	1.33167380928588×10^−2^
*M*(1,0,0)	1.08841503240502×10^0^
*M*(2,0,0)	9.79093689335407×10^−2^
*A*(0,0,1)	1.33537580655960×10^−1^
*A*(1,0,1)	7.65789029061483×10^−2^
*A*(2,0,1)	3.73742902997137×10^−2^
G(0,1,1)	5.31044255713088×10^−1^
*G*(1,1,1)	1.32418227810710×10^1^
*G*(2,1,1)	6.88863709884594×10^−2^
*D*(0,1,1)	1.14118194976730×10^−2^
*D*(1,1,1)	2.99644035332771×10^−1^
*D*(2,1,1)	8.92155178101449×10^−1^
*M*(0,1,1)	7.55711980917015×10^0^
*M*(1,1,1)	6.58304546585478×10^0^
*M*(2,1,1)	4.33636256393215×10^−3^
*X*	4.06993305645860×10^0^

**Table 4:** Example coefficients of model with two cancer stage mutations and one destabilizing mutation.

Coefficient	Value
*G*(1,0,0)	2.22095885699822×10^−3^
*D*(1,0,0)	1.31378739613141×10^−6^
*M*(0,0,0)	8.12022029775447×10^−4^
*M*(1,0,0)	1.40674010365097×10^−5^
*A*(0,0,1)	2.06668108660923×10^−1^
*A*(1,0,1)	4.57214970326658×10^−3^
G(0,1,1)	1.56644835664010×10^−2^
*G*(1,1,1)	3.16379145991048×10^−4^
*D*(0,1,1)	1.29917705679554×10^0^
*D*(1,1,1)	1.92969737536413×10^−1^
*M*(0,1,1)	9.58173133172697×10^0^
*M*(1,1,1)	2.26339224702545×10^−1^
*X*	2.78141105650539×10^−1^

**Table 5:** Eigenvalues in ascending order of Hessian matrix associated with a model with three cancer stage mutations and one destabilizing mutation (as in Table 3), and with a model with two cancer stage mutations and one destabilizing mutation (as in Table 4).

Number	Eigenvalues (Table 3)	Eigenvalues (Table 4)
1	−1.20726415206490×10^1^	−1.45810346778189×10^0^
2	−4.92487558715060×10^0^	−7.77741441881355×10^−1^
3	−1.11648980088601×10^0^	−2.77127189259301×10^−1^
4	−2.44711976272777×10^−1^	−6.66243518532325×10^−3^
5	−9.84288250086772×10^−2^	−3.53209777682867×10^−4^
6	−1.23814589706358×10^−2^	−2.86471102388267×10^−4^
7	−2.95522329598474×10^−3^	**−9.25930409562877×10^−^^6^**
8	−1.53669876331947×10^−3^	**−1.78637642487767×10^−^^11^**
9	−9.80139032107413×10^−5^	2.74342908757636×10^−4^
10	−3.36238129341872×10^−5^	4.98697524563660×10^−4^
11	−2.14105771381677×10^−6^	1.11215731049368×10^−2^
12	**−1.86967299054058×10^−^^7^**	8.18426507233826×10^−1^
13	**5.01559183858810×10^−^^12^**	1.45195703291853×10^0^
14	9.44044820094881×10^−7^	-
15	4.05661818962605×10^−4^	-
16	1.92220119614334×10^−3^	-
17	1.11042617352459×10^−2^	-
18	1.03277102432191×10^−1^	-
19	1.12667702944003×10^0^	-
20	1.08248991510735×10^1^	-

Non-significant eigenvalues are underlined in bold.

## Discussion

We have shown that in the class of stochastic cancer models incorporating genomic instability developed by Little and Wright [11] the number of identifiable combinations of parameters is at most two less than the number of biological parameters, thereby generalizing previous results of Heidenreich *et al.* [8], [9] and Hanin *et al.* [21], [22] for the two-mutation model, a special case of this model. For the more general genomic-instability cancer model of Little *et al.* [12] the number of identifiable combinations of parameters is at most 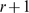 less than the number of biological parameters, where 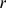 is the number of destabilization types, thereby also generalizing all these results. Numerical evaluations in two special cases (with 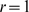) suggest that this bound is tight: a combination of parameters with cardinality two less than the number of biological parameters is of full rank, and so is not redundant.

A weakness of the paper is that one cannot be absolutely sure (because of the uncertainty implicit in any numerical evaluation) that the bound demonstrated by the mathematics of section 3 and Text S1 Section B is sharp. Nevertheless, we have clearly established a maximum number of identifiable parameter combinations. We have also specified particular combinations of identifiable parameters, and these should be used in model fitting to avoid obvious numerical problems, of lack of convergence and absence of a unique set of parameters maximizing the likelihood.

These results have obvious implications for the large number of other quasi-biological cancer models that are special cases of these models, in particular those of Armitage and Doll [13], the two-mutation model [10], the generalized multistage model of Little [14], and a recently developed cancer model of Nowak *et al.* [15] that incorporates genomic instability. It should be noted that the results given here are for the fully stochastic solution of the model, and would not be applicable, for example, to the deterministic approximation of the multistage model of Armitage and Doll [13] that is often employed in applications.

Our results imply that for the general class of cancer models considered here, only certain specific parameter combinations should be estimated in principle, and this is the case whatever the size of the dataset being considered. Whether for complex models for even this theoretically available number of parameters there is useful information is of course uncertain, and may well depend on the particular dataset and on the likely size of the parameters to be estimated. However, fits to a large population-based registry of colon cancer, as recently analysed by Little and Li [16], suggests that, for example, the model with two cancer-stage and one destabilizing mutations can be fitted to the dataset and yields stable parameter estimates for certain combinations of 11 parameters, in accordance with the results of this paper.

## Supporting Information

Text S1 Text S1
(0.42 MB DOC)
