# Supplementary material for: Parameter Identifiability and Redundancy in a General Class of Stochastic Carcinogenesis Models
Source: PLoS One. 2009 Dec 31;4(12):e8520. doi: 10.1371/journal.pone.0008520 (PMC2797326; doi:10.1371/journal.pone.0008520)
Supplement: Text S1 — (0.42 MB DOC) [file pone.0008520.s001.doc]

**Supplementary material A.**

**Derivation of the Kolmogorov forward differential equation for the cancer model of Little *et al.* [12]**

In this Section we derive the Kolmogorov forward differential equation defining the generating function of the cancer model of Little *et al.* [12]. The generating function $\psi$ is given by

$\begin{matrix} \psi(y_{1,0,0},y_{2,0,0},...,y_{k-1,0,0},y_{0,1,1},...,y_{k-1,1,1},y_{0,2,1},y_{1,2,1},...,y_{k-1,m_{r},r},y_{k};t,s) \\ \equiv\psi(t,s) \\ =\sum_{n} y_{1,0,0}^{n_{1,0,0}}\cdot...\cdot y_{k-1,0,0}^{n_{k-1,0,0}}\cdot y_{0,1,1}^{n_{0,1,1}}\cdot...\cdot y_{k-1,1,1}^{n_{k-1,1,1}}\cdot...\cdot y_{k-1,m_{r},r}^{n_{k-1,m_{r},r}}\cdot y_{k}^{n_{k}}\times\\ P[Y_{1,0,0}(t)=n_{1,0,0},...,Y_{k}(t)=n_{k}\left|N(s)=X(0),Y_{1,0,0}(s)=...=Y_{k}(s)=0\right.] \end{matrix}$ (A1)

By differentiating term by term (justified by the absolute convergence of the derivative power series) $\psi$ satisfies:

$$\begin{matrix} \frac{\partial\psi}{\partial t}[t,s]=\sum_{n_{1,0,0},n_{2,0,0},...,n_{k}} y_{1,0,0}^{n_{1,0,0}}\cdot...\cdot y_{k-1,m_{r},r}^{n_{k-1,m_{r},r}}\cdot y_{k}^{n_{k}}\cdot\frac{dP}{dt}[Y_{1,0,0}(t)=n_{1,0,0},...,Y_{k-1,m_{r},r}(t)=n_{k-1,m_{r},r},Y_{k}(t)=n_{k}] \\ =\sum_{n_{1,0,0},n_{2,0,0},...,n_{k}} y_{1,0,0}^{n_{1,0,0}}\cdot...\cdot y_{k-1,m_{r},r}^{n_{k-1,m_{r},r}}\cdot y_{k}^{n_{k}}\cdot X(t)\cdot M(0,0,0)(t)\cdot P[Y_{1,0,0}(t)=n_{1,0,0}-1,...,Y_{k-1,m}(t)=n_{k-1,m_{r},r},Y_{k}(t)=n_{k}]+ \\ \sum_{d=1}^{r} \sum_{n_{1,0,0},n_{2,0,0},...,n_{k}} y_{1,0}^{n_{1,0}}\cdot...\cdot y_{k-1,m_{r},r}^{n_{k-1,m_{r},r}}\cdot y_{k}^{n_{k}}\cdot X(t)\cdot A(0,0,d)(t)\cdot P[Y_{1,0,0}(t)=n_{1,0,0},....,Y_{0,1,d}(t)=n_{0,1,d}-1,...,Y_{k-1,m_{r},r}(t)=n_{k-1,m_{r},r},Y_{k}(t)=n_{k}]- \\ \sum_{n_{1,0,0},n_{2,0,0},...,n_{k}} y_{1,0,0}^{n_{1,0,0}}\cdot...\cdot y_{k-1,m_{r},r}^{n_{k-1,m_{r},r}}\cdot y_{k}^{n_{k}}\cdot X(t)\cdot M(0,0,0)(t)\cdot P[Y_{1,0,0}(t)=n_{1,0,0},...,Y_{k-1,m_{r},r}(t)=n_{k-1,m_{r},r},Y_{k}(t)=n_{k}]- \\ \sum_{d=1}^{r} \sum_{n_{1,0,0},n_{2,0,0},...,n_{k}} y_{1,0,0}^{n_{1,0,0}}\cdot...\cdot y_{k-1,m_{r},r}^{n_{k-1,m_{r},r}}\cdot y_{k}^{n_{k}}\cdot X(t)\cdot A(0,0,d)(t)\cdot P[Y_{1,0,0}(t)=n_{1,0,0},...,Y_{k-1,m_{r},r}(t)=n_{k-1,m_{r},r},Y_{k}(t)=n_{k}]+ \end{matrix}$$

$\begin{matrix} =\psi\cdot[y_{1,0,0}-1]\cdot X(t)\cdot M(0,0,0)(t)+\psi\cdot\sum_{d=1}^{r} [y_{0,1,d}-1]\cdot X(t)\cdot A(0,0,d)(t)+ \\ \sum_{1\leq\alpha\leq k-1} \left[\begin{matrix} -y_{\alpha,0,0}\cdot[D(\alpha,0,0)(t)+G(\alpha,0,0)(t)+M(\alpha,0,0)(t) \\ +\sum_{d'=1}^{r} A(\alpha,0,d')(t)]+y_{\alpha,0,0}^{2}\cdot G(\alpha,0,0)(t)+D(\alpha,0,0)(t) \\ +y_{\alpha,0,0}\cdot y_{\alpha+1,0,0}\cdot M(\alpha,0,0)(t)+\sum_{d'=1}^{r} y_{\alpha,0,0}\cdot y_{\alpha,1,d'}\cdot A(\alpha,0,d')(t) \end{matrix}\right]\cdot\frac{\partial\psi}{\partial y_{\alpha,0,0}} \\ \sum_{\begin{matrix} 0\leq\alpha\leq k-1 \\ 1\leq\beta\leq m_{d} \\ 1\leq d\leq r \end{matrix}} \left[\begin{matrix} -y_{\alpha,\beta,d}\cdot[D(\alpha,\beta,d)(t)+G(\alpha,\beta,d)(t)+M(\alpha,\beta,d)(t) \\ +A(\alpha,\beta,d)(t)]+y_{\alpha,\beta,d}^{2}\cdot G(\alpha,\beta,d)(t)+D(\alpha,\beta,d)(t) \\ +y_{\alpha,\beta,d}\cdot y_{\alpha+1,\beta,d}\cdot M(\alpha,\beta,d)(t)+y_{\alpha,\beta,d}\cdot y_{\alpha,\beta+1,d}\cdot A(\alpha,\beta,d)(t) \end{matrix}\right]\cdot\frac{\partial\psi}{\partial y_{\alpha,\beta,d}} \end{matrix}$ (A2)

**Supplementary material B.**

**Derivation of hazard function in terms of specific parameter combinations**

In this Section we derive the hazard function and show that it can be written in terms of certain combinations of parameters, given in Table 2. The hazard function is defined as:

$h(t)=-\frac{d}{dt}\ln\psi\left(1,1,...,1,0;t,0\right)$ (B1)

where

$\begin{matrix} \psi(y_{1,0,0},y_{2,0,0},...,y_{k-1,0,0},y_{0,1,1},...,y_{k-1,1,1},y_{0,2,1},y_{1,2,1},...,y_{k-1,m_{r},r},y_{k};t,0) \\ \equiv\psi(t) \\ =\sum_{n} y_{1,0,0}^{n_{1,0,0}}\cdot...\cdot y_{k-1,0,0}^{n_{k-1,0,0}}\cdot y_{0,1,1}^{n_{0,1,1}}\cdot...\cdot y_{k-1,1,1}^{n_{k-1,1,1}}\cdot...\cdot y_{k-1,m_{r},r}^{n_{k-1,m_{r},r}}\cdot y_{k}^{n_{k}}\times\\ P\left(Y_{1,0,0}(t)=n_{1,0,0},...,Y_{k}(t)=n_{k}\left|N(0)=X(0),Y_{1,0,0}(0)=...=Y_{k}(0)=0\right.\right) \end{matrix}$ (B2)

is the full probability generating function (PGF) starting with cell(s) in the normal compartment at time 0. The number of biological parameters in this specific model is summarized in Table 1.

By straightforward generalizations of material in Little and Wright [11] (given in Text S1 Section A) it is seen that $\psi$ satisfies a Kolmogorov forward equation:

$\begin{matrix} \frac{d\psi}{dt}=\psi\cdot[y_{1,0,0}-1]\cdot X(t)\cdot M(0,0,0)(t)+\psi\cdot\sum_{d=1}^{r} [y_{0,1,d}-1]\cdot X(t)\cdot A(0,0,d)(t)+ \\ \sum_{1\leq\alpha\leq k-1} \left[\begin{matrix} -y_{\alpha,0,0}\cdot[D(\alpha,0,0)(t)+G(\alpha,0,0)(t)+M(\alpha,0,0)(t) \\ +\sum_{d'=1}^{r} A(\alpha,0,d')(t)]+y_{\alpha,0,0}^{2}\cdot G(\alpha,0,0)(t)+D(\alpha,0,0)(t) \\ +y_{\alpha,0,0}\cdot y_{\alpha+1,0,0}\cdot M(\alpha,0,0)(t)+\sum_{d'=1}^{r} y_{\alpha,0,0}\cdot y_{\alpha,1,d'}\cdot A(\alpha,0,d')(t) \end{matrix}\right]\cdot\frac{\partial\psi}{\partial y_{\alpha,0,0}} \\ \sum_{\begin{matrix} 0\leq\alpha\leq k-1 \\ 1\leq\beta\leq m_{d} \\ 1\leq d\leq r \end{matrix}} \left[\begin{matrix} -y_{\alpha,\beta,d}\cdot[D(\alpha,\beta,d)(t)+G(\alpha,\beta,d)(t)+M(\alpha,\beta,d)(t) \\ +A(\alpha,\beta,d)(t)]+y_{\alpha,\beta,d}^{2}\cdot G(\alpha,\beta,d)(t)+D(\alpha,\beta,d)(t) \\ +y_{\alpha,\beta,d}\cdot y_{\alpha+1,\beta,d}\cdot M(\alpha,\beta,d)(t)+y_{\alpha,\beta,d}\cdot y_{\alpha,\beta+1,d}\cdot A(\alpha,\beta,d)(t) \end{matrix}\right]\cdot\frac{\partial\psi}{\partial y_{\alpha,\beta,d}} \end{matrix}$ (B3)

with the conventions that $y_{\alpha,\beta,d}\equiv0$ for $\beta>m_{d}$, $A(\alpha,\beta,d)\equiv0$ for $\beta\geq m_{d}$ and $y_{k,\beta,d}\equiv y_{k}$ for all defined $\beta$ and $d$. We solve the equation by means of Cauchy’s method of characteristics. Suppose $y_{\alpha,\beta,d}\equiv y_{\alpha,\beta,d}(u)$ and $t\equiv t(u)$, then $\psi\equiv\psi(y_{\alpha,\beta,d}(u),t(u))$. This implies that:

$\begin{matrix} \frac{\partial\psi}{\partial u}=\frac{\partial\psi}{\partial t}\cdot\frac{dt}{du}+\frac{\partial\psi}{\partial y_{k}}\cdot\frac{dy_{k}}{du}+\sum_{1\leq\alpha\leq k-1} \frac{\partial\psi}{\partial y_{\alpha,0,0}}\cdot\frac{dy_{\alpha,0,0}}{du}+\sum_{\begin{matrix} 0\leq\alpha\leq k-1 \\ 1\leq\beta\leq m_{d} \\ 1\leq d\leq r \end{matrix}} \frac{\partial\psi}{\partial y_{\alpha,\beta,d}}\cdot\frac{dy_{\alpha,\beta,d}}{du} \\ =\frac{dt}{du}\cdot\left[\begin{matrix} \psi\cdot[y_{1,0,0}-1]\cdot X(t)\cdot M(0,0,0)(t)+\psi\cdot\sum_{d=1}^{r} [y_{0,1,d}-1]\cdot X(t)\cdot A(0,0,d)(t)+ \\ \sum_{1\leq\alpha\leq k-1} \left[\begin{matrix} -y_{\alpha,0,0}\cdot[D(\alpha,0,0)(t)+G(\alpha,0,0)(t)+M(\alpha,0,0)(t) \\ +\sum_{d'=1}^{r} A(\alpha,0,d')(t)]+y_{\alpha,0,0}^{2}\cdot G(\alpha,0,0)(t)+D(\alpha,0,0)(t) \\ +y_{\alpha,0,0}\cdot y_{\alpha+1,0,0}\cdot M(\alpha,0,0)(t)+\sum_{d'=1}^{r} y_{\alpha,0,0}\cdot y_{\alpha,1,d'}\cdot A(\alpha,0,d')(t) \end{matrix}\right]\cdot\frac{\partial\psi}{\partial y_{\alpha,0,0}} \\ \sum_{\begin{matrix} 0\leq\alpha\leq k-1 \\ 1\leq\beta\leq m_{d} \\ 1\leq d\leq r \end{matrix}} \left[\begin{matrix} -y_{\alpha,\beta,d}\cdot[D(\alpha,\beta,d)(t)+G(\alpha,\beta,d)(t)+M(\alpha,\beta,d)(t) \\ +A(\alpha,\beta,d)(t)]+y_{\alpha,\beta,d}^{2}\cdot G(\alpha,\beta,d)(t)+D(\alpha,\beta,d)(t) \\ +y_{\alpha,\beta,d}\cdot y_{\alpha+1,\beta,d}\cdot M(\alpha,\beta,d)(t)+y_{\alpha,\beta,d}\cdot y_{\alpha,\beta+1,d}\cdot A(\alpha,\beta,d)(t) \end{matrix}\right]\cdot\frac{\partial\psi}{\partial y_{\alpha,\beta,d}} \end{matrix}\right] \\ +\frac{\partial\psi}{\partial y_{k}}\cdot\frac{dy_{k}}{du}+\sum_{1\leq\alpha\leq k-1} \frac{\partial\psi}{\partial y_{\alpha,0,0}}\cdot\frac{dy_{\alpha,0,0}}{du}+\sum_{\begin{matrix} 0\leq\alpha\leq k-1 \\ 1\leq\beta\leq m_{d} \\ 1\leq d\leq r \end{matrix}} \frac{\partial\psi}{\partial y_{\alpha,\beta,d}}\cdot\frac{dy_{\alpha,\beta,d}}{du} \end{matrix}$ (B4)

A solution is therefore given by:

$\frac{\partial\psi}{\partial u}=\psi\cdot[y_{1,0,0}(u)-1]\cdot X(u)\cdot M(0,0,0)(u)+\psi\cdot\sum_{d=1}^{r} [y_{0,1,d}(u)-1]\cdot X(u)\cdot A(0,0,d)(u)$ (B5)

$\frac{\partial t}{\partial u}=1$ (B6)

$\frac{\partial y_{k}}{\partial u}=0$ (B7)

and for $d=0$:

$\begin{matrix} \frac{dy_{\alpha,0,0}}{du}=y_{\alpha,0,0}\cdot[D(\alpha,0,0)(t)+G(\alpha,0,0)(t)+M(\alpha,0,0)(t) \\ +\sum_{d'=1}^{r} A(\alpha,0,d')(t)]-y_{\alpha,0,0}^{2}\cdot G(\alpha,0,0)(t)-D(\alpha,0,0)(t) \\ -y_{\alpha,0,0}\cdot y_{\alpha+1,0,0}\cdot M(\alpha,0,0)(t)-\sum_{d'=1}^{r} y_{\alpha,0,0}\cdot y_{\alpha,1,d'}\cdot A(\alpha,0,d')(t) \end{matrix}$ (B8)

while for $d\neq0$:

$\begin{matrix} \frac{dy_{\alpha,\beta,d}}{du}=y_{\alpha,\beta,d}\cdot[D(\alpha,\beta,d)(t)+G(\alpha,\beta,d)(t)+M(\alpha,\beta,d)(t) \\ +A(\alpha,\beta,d)(t)]-y_{\alpha,\beta,d}^{2}\cdot G(\alpha,\beta,d)(t)-D(\alpha,\beta,d)(t) \\ -y_{\alpha,\beta,d}\cdot y_{\alpha+1,\beta,d}\cdot M(\alpha,\beta,d)(t)-y_{\alpha,\beta,d}\cdot y_{\alpha,\beta+1,d}\cdot A(\alpha,\beta,d)(t) \end{matrix}$ (B9)

For the hazard, a solution is required for $\psi(1,1,1,...,1,0;t,0)$, i.e., $\psi(y_{1,0,0}=1,y_{2,0,0}=1,y_{3,0,0}=1,...,y_{k-1,m_{r},r}=1,y_{k}=0;t,s=0)$, so that a particular characteristic must have the boundary value $y_{\alpha,\beta,d}(t)=1$ and $y_{k}(t)=0$ (implying by (B7)$y_{k}(u)\equiv0$), so that $y_{\alpha,\beta,d}(u)$ is a function of both $u$ and $t$, i.e., $y_{\alpha,\beta,d}(u)\equiv y_{\alpha,\beta,d}(u,t)$. Integrating (B5) over $u\in[0,t]$ yields

$\psi(t)=\exp\left\{\int_{0}^{t} \left[\begin{matrix} \left\{y_{1,0,0}(u,t)\right\}\cdot X(u)\cdot M(0,0,0)(u) \\ +\sum_{d=1}^{r} \left\{y_{0,1,d}(u,t)-1\right\}\cdot X(u)\cdot A(0,0,d)(u) \end{matrix}\right] du \right\}$ (B10)

Assume now that the model parameters $G(\alpha,\beta,d)(t)$, $D(\alpha,\beta,d)(t)$, $M(\alpha,\beta,d)(t)$, $A(\alpha,\beta,d)(t)$ and $X(t)$ are constant over time. By substituting $z_{\alpha,\beta,d}(u,t)=[y_{\alpha,\beta,d}(u,t)-1]\cdot G(\alpha,\beta,d)$ into (B8) and (B9), the following can be obtained

$\left\{\begin{matrix} \begin{matrix} \frac{dz_{\alpha,\beta,d}}{ds}=-z_{\alpha,\beta,d}^{2}+z_{\alpha,\beta,d}\cdot N\left[\alpha,\beta,d,z_{\alpha,\beta+1,d},z_{\alpha+1,\beta,d}\right] \\ +P\left[\alpha,\beta,d,z_{\alpha,\beta+1,d},z_{\alpha+1,\beta,d}\right] \end{matrix} & when & d\neq0 \\ \begin{matrix} \frac{dz_{\alpha,0,0}}{ds}=-z_{\alpha,0,0}^{2}+z_{\alpha,0,0}\cdot N'\left[\alpha,z_{\alpha,1,1},\cdots,z_{\alpha,1,r},z_{\alpha+1,0,0}\right] \\ +P'\left[\alpha,z_{\alpha,1,1},\cdots,z_{\alpha,1,r},z_{\alpha+1,0,0}\right] \end{matrix} & when & d=0 \end{matrix}\right.$ (B11)

where

$N\left[\alpha,\beta,d,v,w\right]=\left\{\begin{matrix} \begin{matrix} D(\alpha,\beta,d)-G(\alpha,\beta,d)-w\cdot\frac{M(\alpha,\beta,d)}{G(\alpha+1,\beta,d)} \\ -v\cdot\frac{A(\alpha,\beta,d)}{G(\alpha,\beta+1,d)} \\ \end{matrix} & (\alpha<k-1,\beta<m_{d}) \\ \begin{matrix} D(\alpha,\beta,d)-G(\alpha,\beta,d)+M(\alpha,\beta,d) \\ -v\cdot\frac{A(\alpha,\beta,d)}{G(\alpha,\beta+1,d)} \\ \end{matrix} & (\alpha=k-1,\beta<m_{d}) \\ \begin{matrix} D(\alpha,\beta,d)-G(\alpha,\beta,d)-w\cdot\frac{M(\alpha,\beta,d)}{G(\alpha+1,\beta,d)} \\ \end{matrix} & (\alpha<k-1,\beta=m_{d}) \\ D(\alpha,\beta,d)-G(\alpha,\beta,d)+M(\alpha,\beta,d) & (\alpha=k-1,\beta=m_{d}) \end{matrix}\right.$ (B12)

$P\left[\alpha,\beta,d,v,w\right]=\left\{\begin{matrix} \begin{matrix} -G(\alpha,\beta,d)\cdot\left[w\cdot\frac{M(\alpha,\beta,d)}{G(\alpha+1,\beta,d)}+v\cdot\frac{A(\alpha,\beta,d)}{G(\alpha,\beta+1,d)}\right] \\ \end{matrix} & (\alpha<k-1,\beta<m_{d}) \\ \begin{matrix} G(\alpha,\beta,d)\cdot\left[M(\alpha,\beta,d)-v\cdot\frac{A(\alpha,\beta,d)}{G(\alpha,\beta+1,d)}\right] \\ \end{matrix} & (\alpha=k-1,\beta<m_{d}) \\ \begin{matrix} -w\cdot\frac{G(\alpha,\beta,d)\cdot M(\alpha,\beta,d)}{G(\alpha+1,\beta,d)} \\ \end{matrix} & (\alpha<k-1,\beta=m_{d}) \\ G(\alpha,\beta,d)\cdot M(\alpha,\beta,d) & (\alpha=k-1,\beta=m_{d}) \end{matrix}\right.$ (B13)

$N'\left[\alpha,v_{1},\cdots,v_{r},w\right]=\left\{\begin{matrix} \begin{matrix} D(\alpha,0,0)-G(\alpha,0,0)-w\cdot\frac{M(\alpha,0,0)}{G(\alpha+1,0,0)} \\ -\sum_{d=1}^{r} v_{d}\cdot\frac{A(\alpha,0,d)}{G(\alpha,1,d)} \\ \end{matrix} & \left(0<\alpha<k-1\right) \\ \begin{matrix} D(\alpha,0,0)-G(\alpha,0,0)+M(\alpha,0,0) \\ -\sum_{d=1}^{r} v_{d}\cdot\frac{A(\alpha,0,d)}{G(\alpha,1,d)} \end{matrix} & \left(\alpha=k-1\right) \end{matrix}\right.$ (B14)

and

$P'\left[\alpha,v_{1},\cdots,v_{r},w\right]=\left\{\begin{matrix} \begin{matrix} -G(\alpha,0,0)\cdot\left[w\cdot\frac{M(\alpha,0,0)}{G(\alpha+1,0,0)}+\sum_{d=1}^{r} v_{d}\cdot\frac{A(\alpha,0,d)}{G(\alpha,1,d)}\right] \\ \end{matrix} & (0<\alpha<k-1) \\ G(\alpha,0,0)\cdot\left[M(\alpha,0,0)-\sum_{d=1}^{r} v_{d}\cdot\frac{A(\alpha,0,d)}{G(\alpha,1,d)}\right] & (\alpha=k-1) \end{matrix}\right.$ (B15)

Likewise, with the same trick, (B10) can be rewritten as

$\psi(t)=\exp\left\{\int_{0}^{t} z_{1,0,0}(s,t)\cdot\frac{X\cdot M(0,0,0)}{G(1,0,0)}+\sum_{d=1}^{r} z_{0,1,d}(s,t)\cdot\frac{X\cdot A(0,0,d)}{G(0,1,d)} ds\right\}$ (B16)

**Supplementary material C.**

**Specification of embedded exponential family model**

In this Section we outline the specification of an embedding of a stochastic cancer model in a general class of statistical models, the so-called exponential family [23]. This is often done in fitting cancer models to epidemiological and biological data (e.g., see references [11, 12, 16]). Recall that a model is a member of the exponential family if the observed data $x=(x_{l})_{l=1}^{n}\in\sum^{n}$ is such that the log-likelihood is given by $L(x|\theta)=\sum_{l=1}^{n} \left[\frac{x_{l}\varsigma_{l}-b(\varsigma_{l})}{a(\varphi)}+c(x_{l},\varphi)\right]$ for some functions $a(\varphi),b(\varsigma),c(x,\varphi)$. We assume that the natural parameters $\varsigma_{l}=\varsigma_{l}[(\theta_{i})_{i=1}^{p},z_{l}]$ are functions of the model parameters $(\theta_{i})_{i=1}^{p}$ and some auxiliary data $(z_{l})_{l=1}^{n}$, and that $\mu_{l}=b'(\varsigma_{l}[(\theta_{i})_{i=1}^{p},z_{l}])=z_{l}\cdot h[(\theta_{i})_{i=1}^{p},y_{l}]$. Here $h[(\theta_{i})_{i=1}^{p},y_{l}]$ is the cancer hazard function (for example, that of Little *et al.* [12] specified in Sections 2 and 3 of the main text and in Text S1 Section B), $(y_{l})_{l=1}^{n}$ are some further auxiliary data, and we assume that the $(z_{l})_{l=1}^{n}$ are all non-zero. [Note: this is not necessarily a generalized linear model (GLM).] In this case it is seen that

$\frac{\partial^{2}L(x|\theta)}{\partial\theta_{i}\partial\theta_{j}}=\sum_{l=1}^{n} \left[\begin{matrix} \frac{[x_{l}-b'(\varsigma_{l})]z_{l}}{a(\varphi)b''(\varsigma_{l})}\frac{\partial^{2}h(\theta,y_{l})}{\partial\theta_{i}\partial\theta_{j}} \\ -\frac{z_{l}^{2}}{a(\varphi)}\frac{\partial h(\theta,y_{l})}{\partial\theta_{i}}\frac{\partial h(\theta,y_{l})}{\partial\theta_{j}}\left\{\frac{[b''(\varsigma_{l})]^{2}+b'''(\varsigma_{l})[x_{l}-b'(\varsigma_{l})]}{[b''(\varsigma_{l})]^{3}}\right\} \end{matrix}\right]$ (C1)

so that the Fisher information matrix is given by

$I(\theta)_{ij}=-E_{\theta}\left[\frac{\partial^{2}L(x|\theta)}{\partial\theta_{i}\partial\theta_{j}}\right]=\frac{1}{a(\varphi)}\sum_{l=1}^{n} \frac{z_{l}^{2}}{b''(\varsigma_{l})}\frac{\partial h(\theta,y_{l})}{\partial\theta_{i}}\frac{\partial h(\theta,y_{l})}{\partial\theta_{j}}$ (C2)

**Supplementary material D.**

**Derivation of system of differential equations defining the Hessian of the hazard function for the cancer model of Little and Wright [11] and Little *et al.* [12]**

In this Section we derive the set of differential equations defining the Hessian (with respect to the model parameters) for the cancer model of Little and Wright [11] and Little *et al.* [12] in the case when all model parameters are constant. For simplicity we present only the derivation for the simpler model of Little and Wright [11]; the derivation for the more complex model of Little *et al.* [12] is straightforward but lengthy. This allows us to drop the final identifying label in each of $G(\alpha,\beta,d),D(\alpha,\beta,d),M(\alpha,\beta,d),A(\alpha,\beta,d)$, which we will henceforth write as $G(\alpha,\beta),D(\alpha,\beta),M(\alpha,\beta),A(\alpha,\beta)$, respectively. The hazard function of the cancer model with $k$ cancer-stage mutations and $m$ destabilizing mutations developed by Little and Wright [11] may be written as:

$h(t)=-\int_{0}^{t} \left\{\frac{\partial\varphi_{1,0}[t,s]}{\partial t}\cdot M(0,0)+\frac{\partial\varphi_{0,1}[t,s]}{\partial t}\cdot A(0,0)\right\}\cdot Xds$ (D1)

where the PGFs $\varphi_{i,j}$ also satisfy the following Kolmogorov backward equations (for $0\leq i\leq k-1$, $0\leq j\leq m$, $(i,j)\neq(0,0)$):

$\begin{matrix} \frac{\partial\varphi_{i,j}}{\partial s}[t,s]=[D(i,j)+G(i,j)+M(i,j)+A(i,j)]\cdot\varphi_{i,j}[t,s] \\ -G(i,j)\cdot\varphi_{i,j}[t,s]^{2}-M(i,j)\cdot\varphi_{i,j}[t,s]\cdot\varphi_{i+1,j}[t,s] \\ -A(i,j)\cdot\varphi_{i,j}[t,s]\cdot\varphi_{i,j+1}[t,s]-D(i,j) \end{matrix}$ (D2)

Differentiating (C1) gives:

$\begin{matrix} \frac{\partial h(t)}{\partial X}=-\int_{0}^{t} \left\{\frac{\partial\varphi_{1,0}[t,s]}{\partial t}\cdot M(0,0)+\frac{\partial\varphi_{0,1}[t,s]}{\partial t}\cdot A(0,0)\right\}ds \\ =[1-\varphi_{1,0}[t,0]]\cdot M(0,0)+[1-\varphi_{0,1}[t,0]]\cdot A(0,0) \end{matrix}$ (D3)

$\frac{\partial h(t)}{\partial M(0,0)}=-\int_{0}^{t} \frac{\partial\varphi_{1,0}[t,s]}{\partial t}\cdot Xds=[1-\varphi_{1,0}[t,0]]\cdot X$ (D4)

$\frac{\partial h(t)}{\partial A(0,0)}=-\int_{0}^{t} \frac{\partial\varphi_{0,1}[t,s]}{\partial t}\cdot Xds=[1-\varphi_{0,1}[t,0]]\cdot X$ (D5)

and for all other model parameters, $\beta_{k}$:

$\begin{matrix} \frac{\partial h(t)}{\partial\beta_{k}}=-\int_{0}^{t} \left\{\frac{\partial^{2}\varphi_{1,0}[t,s]}{\partial\beta_{k}\partial t}\cdot M(0,0)+\frac{\partial^{2}\varphi_{0,1}[t,s]}{\partial\beta_{k}\partial t}\cdot A(0,0)\right\}Xds \\ =-\frac{\partial\varphi_{1,0}[t,0]}{\partial\beta_{k}}\cdot M(0,0)-\frac{\partial\varphi_{0,1}[t,0]}{\partial\beta_{k}}\cdot A(0,0) \end{matrix}$ (D6)

Likewise, we can evaluate the second derivatives by differentiating (D3)-(D5) further:

$\frac{\partial^{2}h(t)}{\partial X\partial M(0,0)}=1-\varphi_{1,0}[t,0]$ (D7)

$\frac{\partial^{2}h(t)}{\partial X\partial A(0,0)}=1-\varphi_{0,1}[t,0]$ (D8)

$\frac{\partial^{2}h(t)}{\partial^{2}M(0,0)}=\frac{\partial^{2}h(t)}{\partial^{2}A(0,0)}=\frac{\partial^{2}h(t)}{\partial A(0,0)\partial M(0,0)}=0$ (D9)

and for all model parameters, $\beta_{k},\beta_{l}\notin\left\{X,M(0,0),A(0,0)\right\}$:

$\frac{\partial^{2}h(t)}{\partial\beta_{k}\partial X}=-\frac{\partial\varphi_{1,0}[t,0]}{\partial\beta_{k}}\cdot M(0,0)-\frac{\partial\varphi_{0,1}[t,0]}{\partial\beta_{k}}\cdot A(0,0)$ (D10)

$\frac{\partial^{2}h(t)}{\partial\beta_{k}\partial M(0,0)}=-\frac{\partial\varphi_{1,0}[t,0]}{\partial\beta_{k}}\cdot X$ (D11)

$\frac{\partial^{2}h(t)}{\partial\beta_{k}\partial A(0,0)}=-\frac{\partial\varphi_{0,1}[t,0]}{\partial\beta_{k}}\cdot X$ (D12)

$\frac{\partial^{2}h(t)}{\partial\beta_{k}\partial\beta_{l}}=-\frac{\partial^{2}\varphi_{1,0}[t,0]}{\partial\beta_{k}\partial\beta_{l}}\cdot M(0,0)-\frac{\partial^{2}\varphi_{0,1}[t,0]}{\partial\beta_{k}\partial\beta_{l}}\cdot A(0,0)$ (D13)

We can evaluate $\frac{\partial\varphi_{i,j}[t,s]}{\partial\beta_{k}}$ by differentiating (D2), for $\beta_{k}\notin\left\{D(i,j),G(i,j),M(i,j),A(i,j)\right\}$:

$\begin{matrix} \frac{\partial^{2}\varphi_{i,j}}{\partial\beta_{k}\partial s}[t,s]=[D(i,j)+G(i,j)+M(i,j)+A(i,j)]\cdot\frac{\partial\varphi_{i,j}}{\partial\beta_{k}}[t,s] \\ -2\cdot G(i,j)\cdot\varphi_{i,j}[t,s]\cdot\frac{\partial\varphi_{i,j}}{\partial\beta_{k}}[t,s]-M(i,j)\cdot\left[\frac{\partial\varphi_{i,j}}{\partial\beta_{k}}[t,s]\cdot\varphi_{i+1,j}[t,s]+\frac{\partial\varphi_{i+1,j}}{\partial\beta_{k}}[t,s]\cdot\varphi_{i,j}[t,s]\right] \\ -A(i,j)\cdot\left[\frac{\partial\varphi_{i,j}}{\partial\beta_{k}}[t,s]\cdot\varphi_{i,j+1}[t,s]+\frac{\partial\varphi_{i,j+1}}{\partial\beta_{k}}[t,s]\cdot\varphi_{i,j}[t,s]\right] \\ =\Omega_{i,j}\left[\beta_{k},t,s\right] \end{matrix}$ (D14)

with appropriate initial conditions (discussed later). For $\beta_{k}\in\left\{D(i,j),G(i,j),M(i,j),A(i,j)\right\}$ we have:

$\frac{\partial^{2}\varphi_{i,j}}{\partial\beta_{k}\partial s}[t,s]=\left\{\begin{matrix} \Omega_{i,j}\left[\beta_{k},t,s\right]+\varphi_{i,j}[t,s]-1 \beta_{k}=D(i,j) \\ \Omega_{i,j}\left[\beta_{k},t,s\right]+\varphi_{i,j}[t,s]-\varphi_{i,j}[t,s]^{2} \beta_{k}=G(i,j) \\ \Omega_{i,j}\left[\beta_{k},t,s\right]+\varphi_{i,j}[t,s]-\varphi_{i,j}[t,s]\varphi_{i+1,j}[t,s] \beta_{k}=M(i,j) \\ \Omega_{i,j}\left[\beta_{k},t,s\right]+\varphi_{i,j}[t,s]-\varphi_{i,j}[t,s]\varphi_{i,j+1}[t,s] \beta_{k}=A(i,j) \end{matrix}\right.$ (D15)

Likewise, we can evaluate $\frac{\partial^{2}h(t)}{\partial\beta_{k}\partial\beta_{l}}$ by differentiating (D14), for $\beta_{k},\beta_{l}\notin\left\{D(i,j),G(i,j),M(i,j),A(i,j)\right\}$:

$\begin{matrix} \frac{\partial^{3}\varphi_{i,j}}{\partial\beta_{k}\partial\beta_{l}\partial s}[t,s]=[D(i,j)+G(i,j)+M(i,j)+A(i,j)]\cdot\frac{\partial^{2}\varphi_{i,j}}{\partial\beta_{k}\partial\beta_{l}}[t,s] \\ -2\cdot G(i,j)\cdot\left[\frac{\partial\varphi_{i,j}}{\partial\beta_{k}}[t,s]\cdot\frac{\partial\varphi_{i,j}}{\partial\beta_{l}}[t,s]+\varphi_{i,j}[t,s]\cdot\frac{\partial^{2}\varphi_{i,j}}{\partial\beta_{k}\partial\beta_{l}}[t,s]\right] \\ -M(i,j)\cdot\left[\begin{matrix} \frac{\partial^{2}\varphi_{i,j}}{\partial\beta_{k}\partial\beta_{l}}[t,s]\cdot\varphi_{i+1,j}[t,s]+\frac{\partial\varphi_{i,j}}{\partial\beta_{k}}[t,s]\cdot\frac{\partial\varphi_{i+1,j}}{\partial\beta_{l}}[t,s]+\frac{\partial\varphi_{i,j}}{\partial\beta_{l}}[t,s]\cdot\frac{\partial\varphi_{i+1,j}}{\partial\beta_{k}}[t,s]+ \\ \frac{\partial^{2}\varphi_{i+1,j}}{\partial\beta_{k}\partial\beta_{l}}[t,s]\cdot\varphi_{i,j}[t,s] \end{matrix}\right] \\ -A(i,j)\cdot\left[\begin{matrix} \frac{\partial^{2}\varphi_{i,j}}{\partial\beta_{k}\partial\beta_{l}}[t,s]\cdot\varphi_{i,j+1}[t,s]+\frac{\partial\varphi_{i,j}}{\partial\beta_{k}}[t,s]\cdot\frac{\partial\varphi_{i,j+1}}{\partial\beta_{l}}[t,s]+\frac{\partial\varphi_{i,j}}{\partial\beta_{l}}[t,s]\cdot\frac{\partial\varphi_{i,j+1}}{\partial\beta_{k}}[t,s]+ \\ \frac{\partial^{2}\varphi_{i,j+1}}{\partial\beta_{k}\partial\beta_{l}}[t,s]\cdot\varphi_{i,j}[t,s] \end{matrix}\right] \\ =\Psi_{i,j}\left[\beta_{k},\beta_{l},t,s\right] \end{matrix}$ (D16)

For $\beta_{k}\notin\left\{D(i,j),G(i,j),M(i,j),A(i,j)\right\}$ we have:

$\frac{\partial^{3}\varphi_{i,j}}{\partial\beta_{k}\partial\beta_{l}\partial s}[t,s]=\left\{\begin{matrix} \Psi_{i,j}\left[\beta_{k},\beta_{l},t,s\right]+\frac{\partial\varphi_{i,j}[t,s]}{\partial\beta_{k}} \beta_{l}=D(i,j) \\ \begin{matrix} \Psi_{i,j}\left[\beta_{k},\beta_{l},t,s\right]+\frac{\partial\varphi_{i,j}[t,s]}{\partial\beta_{k}} \beta_{l}=G(i,j) \\ -2\cdot\frac{\partial\varphi_{i,j}[t,s]}{\partial\beta_{k}}\cdot\varphi_{i,j}[t,s] \end{matrix} \\ \begin{matrix} \Psi_{i,j}\left[\beta_{k},\beta_{l},t,s\right]+\frac{\partial\varphi_{i,j}[t,s]}{\partial\beta_{k}}-\frac{\partial\varphi_{i,j}[t,s]}{\partial\beta_{k}}\cdot\varphi_{i+1,j}[t,s] \beta_{l}=M(i,j) \\ -\frac{\partial\varphi_{i+1,j}[t,s]}{\partial\beta_{k}}\cdot\varphi_{i,j}[t,s] \end{matrix} \\ \begin{matrix} \Psi_{i,j}\left[\beta_{k},\beta_{l},t,s\right]+\frac{\partial\varphi_{i,j}[t,s]}{\partial\beta_{k}}-\frac{\partial\varphi_{i,j}[t,s]}{\partial\beta_{k}}\cdot\varphi_{i,j+1}[t,s] \beta_{l}=A(i,j) \\ -\frac{\partial\varphi_{i,j+1}[t,s]}{\partial\beta_{k}}\cdot\varphi_{i,j}[t,s] \end{matrix} \end{matrix}\right.$ (D17)

Finally, we have that:

$\frac{\partial^{3}\varphi_{i,j}}{\partial D(i,j)^{2}\partial s}[t,s]=\Psi_{i,j}\left[D(i,j),D(i,j),t,s\right]+2\cdot\frac{\partial\varphi_{i,j}[t,s]}{\partial D(i,j)}$ (D18)

$\frac{\partial^{3}\varphi_{i,j}}{\partial D(i,j)\partial G(i,j)\partial s}[t,s]=\Psi_{i,j}\left[D(i,j),G(i,j),t,s\right]+\frac{\partial\varphi_{i,j}[t,s]}{\partial D(i,j)}+\frac{\partial\varphi_{i,j}[t,s]}{\partial G(i,j)}-2\cdot\frac{\partial\varphi_{i,j}[t,s]}{\partial D(i,j)}\cdot\varphi_{i,j}[t,s]$ (D19)

$\frac{\partial^{3}\varphi_{i,j}}{\partial D(i,j)\partial M(i,j)\partial s}[t,s]=\Psi_{i,j}\left[D(i,j),M(i,j),t,s\right]+\frac{\partial\varphi_{i,j}[t,s]}{\partial D(i,j)}+\frac{\partial\varphi_{i,j}[t,s]}{\partial M(i,j)}-\frac{\partial\varphi_{i,j}[t,s]}{\partial D(i,j)}\cdot\varphi_{i+1,j}[t,s]$ (D20)

$\frac{\partial^{3}\varphi_{i,j}}{\partial D(i,j)\partial A(i,j)\partial s}[t,s]=\Psi_{i,j}\left[D(i,j),A(i,j),t,s\right]+\frac{\partial\varphi_{i,j}[t,s]}{\partial D(i,j)}+\frac{\partial\varphi_{i,j}[t,s]}{\partial A(i,j)}-\frac{\partial\varphi_{i,j}[t,s]}{\partial D(i,j)}\cdot\varphi_{i,j+1}[t,s]$ (D21)

$\frac{\partial^{3}\varphi_{i,j}}{\partial G(i,j)^{2}\partial s}[t,s]=\Psi_{i,j}\left[G(i,j),G(i,j),t,s\right]+2\cdot\frac{\partial\varphi_{i,j}[t,s]}{\partial G(i,j)}-4\cdot\frac{\partial\varphi_{i,j}[t,s]}{\partial G(i,j)}\cdot\varphi_{i,j}[t,s]$ (D22)

$\begin{matrix} \frac{\partial^{3}\varphi_{i,j}}{\partial G(i,j)\partial M(i,j)\partial s}[t,s]=\Psi_{i,j}\left[G(i,j),M(i,j),t,s\right]+\frac{\partial\varphi_{i,j}[t,s]}{\partial G(i,j)}+\frac{\partial\varphi_{i,j}[t,s]}{\partial M(i,j)}-2\cdot\frac{\partial\varphi_{i,j}[t,s]}{\partial M(i,j)}\cdot\varphi_{i,j}[t,s] \\ -\frac{\partial\varphi_{i,j}[t,s]}{\partial G(i,j)}\cdot\varphi_{i+1,j}[t,s] \end{matrix}$ (D23)

$$\begin{matrix} \frac{\partial^{3}\varphi_{i,j}}{\partial G(i,j)\partial A(i,j)\partial s}[t,s]=\Psi_{i,j}\left[G(i,j),A(i,j),t,s\right]+\frac{\partial\varphi_{i,j}[t,s]}{\partial G(i,j)}+\frac{\partial\varphi_{i,j}[t,s]}{\partial A(i,j)}-2\cdot\frac{\partial\varphi_{i,j}[t,s]}{\partial A(i,j)}\cdot\varphi_{i,j}[t,s] \\ -\frac{\partial\varphi_{i,j}[t,s]}{\partial G(i,j)}\cdot\varphi_{i,j+1}[t,s] \end{matrix}$$

(D24)

$\frac{\partial^{3}\varphi_{i,j}}{\partial M(i,j)^{2}\partial s}[t,s]=\Psi_{i,j}\left[M(i,j),M(i,j),t,s\right]+2\cdot\frac{\partial\varphi_{i,j}[t,s]}{\partial M(i,j)}-2\cdot\frac{\partial\varphi_{i,j}[t,s]}{\partial M(i,j)}\cdot\varphi_{i+1,j}[t,s]$ (D25)

$$\begin{matrix} \frac{\partial^{3}\varphi_{i,j}}{\partial M(i,j)\partial A(i,j)\partial s}[t,s]=\Psi_{i,j}\left[M(i,j),A(i,j),t,s\right]+\frac{\partial\varphi_{i,j}[t,s]}{\partial M(i,j)}+\frac{\partial\varphi_{i,j}[t,s]}{\partial A(i,j)}-\frac{\partial\varphi_{i,j}[t,s]}{\partial M(i,j)}\cdot\varphi_{i,j+1}[t,s] \\ -\frac{\partial\varphi_{i,j}[t,s]}{\partial A(i,j)}\cdot\varphi_{i+1,j}[t,s] \end{matrix}$$

(D26)

$\frac{\partial^{3}\varphi_{i,j}}{\partial A(i,j)^{2}\partial s}[t,s]=\Psi_{i,j}\left[A(i,j),A(i,j),t,s\right]+2\cdot\frac{\partial\varphi_{i,j}[t,s]}{\partial A(i,j)}-2\cdot\frac{\partial\varphi_{i,j}[t,s]}{\partial A(i,j)}\cdot\varphi_{i,j+1}[t,s]$ (D27)

As in Little and Wright [11], the following boundary conditions must be satisfied, for all $i,j,\beta_{k},\beta_{l}$:

$\varphi_{i,j}[t,t]=1 0\leq i\leq k-1$ (D28)

$\frac{\partial\varphi_{i,j}[t,t]}{\partial\beta_{k}}=\frac{\partial^{2}\varphi_{i,j}[t,t]}{\partial\beta_{k}\partial\beta_{l}}=0$ (D29)

This system of ordinary differential equations (in the variable $s$) for $\varphi_{i,j}[t,s]$, $\frac{\partial\varphi_{i,j}[t,s]}{\partial\beta_{k}}$, $\frac{\partial^{2}\varphi_{i,j}[t,s]}{\partial\beta_{k}\partial\beta_{l}}$ were integrated using the Boerlisch-Stoer algorithm with adaptive stepsize control [18]. Very similar results were obtained using a Runge-Kutta integrator with adaptive stepsize control [18].
